# The Differential Proteome of the Probiotic *Lactobacillus acidophilus* NCFM Grown on the Potential Prebiotic Cellobiose Shows Upregulation of Two ***β***-Glycoside Hydrolases

**DOI:** 10.1155/2015/347216

**Published:** 2015-04-19

**Authors:** Gabriella C. van Zanten, Nadja Sparding, Avishek Majumder, Sampo J. Lahtinen, Birte Svensson, Susanne Jacobsen

**Affiliations:** ^1^Department of Food Science, Faculty of Science, University of Copenhagen, Rolighedsvej 26, 1958 Frederiksberg C, Denmark; ^2^Enzyme and Protein Chemistry, Department of Systems Biology, Technical University of Denmark, Søltofts Plads, Building 224, 2800 Kongens Lyngb, Denmark; ^3^DuPont Nutrition and Health, Sokeritehtaantie 20, 02460 Kantvik, Finland

## Abstract

Probiotics, prebiotics, and combinations thereof, that is, synbiotics, are known to exert beneficial health effects in humans; however interactions between pro- and prebiotics remain poorly understood at the molecular level. The present study describes changes in abundance of different proteins of the probiotic bacterium *Lactobacillus acidophilus* NCFM (NCFM) when grown on the potential prebiotic cellobiose as compared to glucose. Cytosolic cell extract proteomes after harvest at late exponential phase of NCFM grown on cellobiose or glucose were analyzed by two dimensional difference gel electrophoresis (2D-DIGE) in the acidic (pH 4–7) and the alkaline (pH 6–11) regions showing a total of 136 spots to change in abundance. Proteins were identified by MS or MS/MS from 81 of these spots representing 49 unique proteins and either increasing 1.5–13.9-fold or decreasing 1.5–7.8-fold in relative abundance. Many of these proteins were associated with energy metabolism, including the cellobiose related glycoside hydrolases phospho-*β*-glucosidase (LBA0881) and phospho-*β*-galactosidase II (LBA0726). The data provide insight into the utilization of the candidate prebiotic cellobiose by the probiotic bacterium NCFM. Several of the upregulated or downregulated identified proteins associated with utilization of cellobiose indicate the presence of carbon catabolite repression and regulation of enzymes involved in carbohydrate metabolism.

## 1. Background

Probiotics are defined as “live microorganisms that when administered in adequate amounts confer a health benefit on the host” [1]. Reported beneficial effects include prevention and reduced severity of respiratory infections [2], modulation of immune responses [3], diminished symptoms of irritable bowel disease [4], and a remedy against antibiotic-associated [4] and infectious diarrhea [5].* Lactobacillus acidophilus* NCFM (NCFM) is a low GC ratio (38.4%), Gram-positive lactic acid bacterium with well-documented probiotic effects [6]. NCFM has a genome of 2.0 Mb with 1,864 predicted open reading frames (ORFs) including different genes and gene loci involved in carbohydrate metabolism [7]. Furthermore, a large number of identified proteins are dedicated to energy metabolism [8]. Noticeably, cDNA microarray analyses of different oligosaccharide transporters support that NCFM is capable of utilizing a wide range of carbohydrates including known prebiotics, such as fructooligosaccharides (FOSs), galactooligosaccharides (GOSs), and potential prebiotics, for example, cellobiose [9, 10].

Prebiotics are food ingredients not digested by the host which selectively stimulate bacterial species of the gut microbiota that confer health benefits, which include stimulation of mineral absorption, improvement of the intestinal barrier, and modulation of immune functions [11]. Since cellobiose (4-O-*β*-d-glucopyranosyl-d-glucose) is not digested in the human upper gastrointestinal tract it is available for bacterial fermentation in the colon [12]. In* in vitro* experiments, in the presence of human fecal bacteria, cellobiose was reported to increase production of short-chain fatty acids (SCFA) [13, 14] which are important for maintenance of epithelial cell homeostasis [15] and possess anti-inflammatory [16] as well as anticarcinogenic effects [17]. In mice, cellobiose thus improved clinical and pathological markers of experimentally induced colitis [18], indicating that cellobiose may exert prebiotic effects also in humans.

A synergistic effect can be achieved when a probiotic and a prebiotic are administered together as a synbiotic [19]. Reported beneficial outcomes include decreased postoperative infection rates [20] and improvements of liver function in patients with liver failure [21]. The combination of NCFM and lactitol is thus found to improve mucosal integrity, intestinal motility, and gut microbiota composition of elderly subjects [22, 23]. A simulated model of the human colon inoculated with fecal bacteria was used to demonstrate the synbiotic potential of NCFM in combination with cellobiose [14]. Recently this combination was demonstrated to increase bifidobacteria and lactobacilli, both regarded as beneficial, in healthy humans [24].

Recently transcriptomic analysis of cellobiose metabolism in* Clostridium acetobutylicum*, a low G+C, Gram-positive bacteria, found in a number of ecological niches such as soil and feces, indicated the presence of multiple cellobiose-inducible operons [25]. Differential transcriptomics and functional genomics have been used to study genes of NCFM involved in uptake of cellobiose [26]; however, molecular responses at the protein level were not explored. Effects of prebiotics on probiotic are not well studied; however, human milk oligosaccharides were recently shown to increase the adhesion of* Bifidobacterium longum* subsp.* infantitis* [27]. Changes in adhesion were correlated to changes at the transcriptional level for genes involved in host interaction, including some encoding so-called moonlighting proteins, that is, proteins having multiple and apparently unrelated activities in different locations in the cell. The present study investigates changes in abundances of individual NCFM proteins as elicited by growth on cellobiose by using 2D-DIGE and mass spectrometry.

## 2. Methods

### 2.1. Growth Conditions

Freeze dried commercial* Lactobacillus acidophilus* NCFM (NCFM; Danisco USA Inc., Madison, US-WI) was revived in semisynthetic medium for lactic acid bacteria (LABSEM) [28] supplemented with 1% (w/v) of either cellobiose or glucose (Sigma-Aldrich, Brøndby, Denmark). To avoid carry-over effects, three cycles of subcultivation were performed (40 mL). Cells in the late exponential phase were harvested by centrifugation (3,200 g, 10 min, 4°C). Growth experiments were done in quadruple. Cells were harvested at OD_600_ 2.0 and 0.7 for glucose and cellobiose, respectably, washed twice in 0.9% NaCl (pH 5.5, 4°C), and centrifuged (10,000 g, 5 min, 4°C). The pellets were dried (2 h, SpeedVac, Savant, SC110A with vacuum unit UVS400A; GMI Inc., Ramsey, US-MN) and stored at −80°C until use.

### 2.2. Preparation and Fluorophore-Labelling of Cytosolic Protein Samples

Extraction of cytosolic proteins was done by mechanical grinding as described [29]. Protein concentrations were determined using the 2D Quant Kit (GE Life Sciences) and samples were stored at −80°C.

The CyDye DIGE Fluor minimal dyes (GE Life Sciences), Cy3 or Cy5 (200 pmol), were used to label aliquots of four biological replicates (50 *μ*g each) interchangeably according to Ettan DIGE System manual (GE Life Sciences) using the dye swapping approach. Internal standards were prepared by mixing protein samples (25 *μ*g from each) and labeling with Cy2 (400 pmol). Samples for analysis at pH 4–7 were mixed with the internal sample and adjusted to 450 *μ*L with rehydration buffer (7 M urea, 2 M thiourea, 3% 3-((3-cholamidopropyl)-dimethylammonio)1-propansulfonate (CHAPS), 1.5% pharmalyte pH 4–7, 1% bis(2-hydroxyethyl) disulfide (HED), 100 mM DTT, and 10 mM Tris-HCl) for immobilized pH gradient (IPG) strips (linear pH 4–7, 24 cm) and incubated in the dark for 30 min at room temperature. For analysis at pH 6–11 samples were mixed as above and the volume was adjusted to 120 *μ*L with rehydration buffer (7 M urea, 2 M thiourea, 3% CHAPS, 1% pharmalyte solution pH 6–11, 2.5% HED, 10% isopropanol, 5% glycerol, and 10 mM Tris-HCl).

### 2.3. 2D-DIGE

For the acidic proteome isoelectric focusing (IEF) was done at a total of 75 kVh (6 h at 30 V, 6 h at 60 V, 1 h at 500 V, 1 h at 1000 V, and 2 h gradient to 8000 V and hold at 8000 V until a minimum of 75 kVh was reached) using IPG strips (pH 4–7, 24 cm, GE Life Sciences) on Ettan IPGphor (GE Life Sciences). For the alkaline region, strips (pH 6–11, 18 cm, GE Life Sciences) were rehydrated in 7 M urea, 2 M thiourea, 3% CHAPS, 1% pharmalyte solution pH 6–11, 2.5% HED, 10% isopropanol, 5% glycerol, and 10 mM Tris-HCl, overnight prior to loading of the sample using the cup-loading method [30, 31]. The IEF was done at a total of 40 kVh (10 min at 30 V, 5 h at 100 V, 5 h at 600 V, 3 h gradient to 4000 V, and 2 h gradient to 8000 V and hold at 8000 V until a minimum of 40 kVh was reached). IPG strips were subsequently equilibrated in 5 mL equilibration buffer (6 M urea, 30% glycerol, 50 mM Tris-HCl pH 8.8, 0.01% bromophenol blue, and 2% SDS) with 1% DTT (15 min) followed by 5 mL equilibration buffer with 2.5% iodoacetamide (15 min). Separation in the second dimension was performed with 12.5% SDS-PAGE gels on Ettan DALT* twelve* Electrophoresis Unit (GE Life Sciences) as previously described [8].

### 2.4. Image Analysis

Immediately after the second dimension DIGE, gels were imaged at excitation/emission wavelengths of Cy2 (488/520 nm), Cy3 (532/580 nm), and Cy5 (633/670 nm) at 100 *μ*m resolution (Typhoon 9410 Variable Mode Imager; GE Life Sciences, Uppsala, Sweden). The obtained images were analyzed using Progenesis SameSpots v 4.0 software (Nonlinear Dynamics Ltd.). Alignment of gel images was performed by automated calculation of 20 manually assigned vectors. Differentially expressed proteins (threshold of ≥ 1.5-fold difference in spot intensity) were chosen based on power calculation of 80% (minimum sample size required to accept the outcome of a statistical test) and the *q*-value was set at 0.05 (*q* ≤ 0.05) giving a false discovery rate of 5% (*P* ≤ 0.05). Prior to spot identification, gels were stained with colloidal Coomassie Brilliant Blue as described previously [32].

### 2.5. In-Gel Digestion and Protein Identification

Spots of varying relative intensity were manually excised and subjected to in-gel tryptic digestion [8]. MS and MS/MS were performed as previously described [8]. Spectra were searched against the NCBInr database for bacteria (20100323, 10606545 sequences, 3615943919 residues) using MASCOT 2.0 software (http://www.matrixscience.com/) integrated with BioTools v3.1 (Bruker-Daltonics). The parameters for the search were monoisotopic peptide mass accuracy of 80 ppm, fragment mass accuracy of 0.7 Da, missed cleavage of maximum one, carbamidomethylation of cysteine, and partial oxidation of methionine. Known keratin and autocatalytic trypsin peaks were removed and the signal to noise ratio was set at 1 : 6. A Mascot score of ≥ 80 (*P* ≤ 0.05) was used to confirm proteins identified by peptide mass fingerprint and should have a minimum of six matched peptides. For MS/MS based identification of proteins a Mascot score of ≥ 40 (*P* ≤ 0.05) was used for each peptide.

## 3. Results

### 3.1. Acidic and Alkaline Differential Proteome of NCFM Grown on Cellobiose

**Figure 1:**
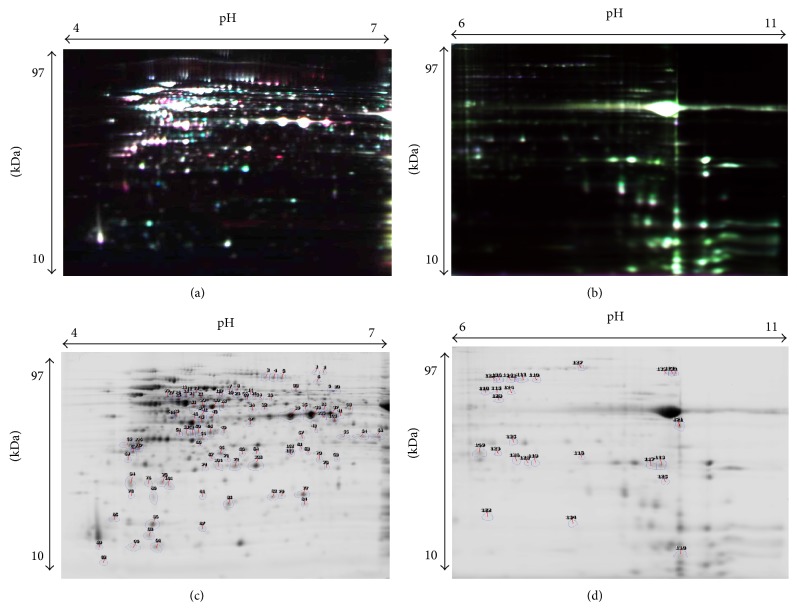
Representative 2D-DIGE fluorescent gel images of* Lactobacillus acidophilus* NCFM soluble cytosolic proteins for (a) the acidic region pH 4–7 and (b) the alkaline region pH 6–11. 2D-DIGE Coomassie Brilliant Blue gel images showing numbers indicating spots picked for identification by mass spectrometry are shown for (c) the acidic region pH 4–7 and (d) the alkaline region pH 6–11.

The present investigation of cytosolic proteomes of NCFM grown on cellobiose, a potential synbiotic combination, and glucose identified a total of 136 differentially abundant spots in 2D-DIGE (Figure 1). Of these 108 (spots 1–108) were in the acidic region (pH 4–7, Figure 1(c)) and 28 spots in the alkaline region (pH 6–11, Figure 1(d)), respectively. This difference is in accordance with the cytosolic proteome distribution of NCFM [8, 29]. Among the 136 spots, 81 showed higher (1.5–13.9-fold) and 55 lower (1.5–7.8-fold) relative abundance. Proteins were identified with confidence by MS and/or MS/MS in 81 of 136 picked spots, representing 49 unique proteins. The 81 spots correspond to 64 and 17 identifications from the acidic and alkaline regions, respectively (Tables S1 and S2). The hypothetical protein LBA0890 (spots 54–56 and 124) was identified both in the acidic and alkaline proteome in the overlapping region (p*I* 6-7).

**Figure 2:**
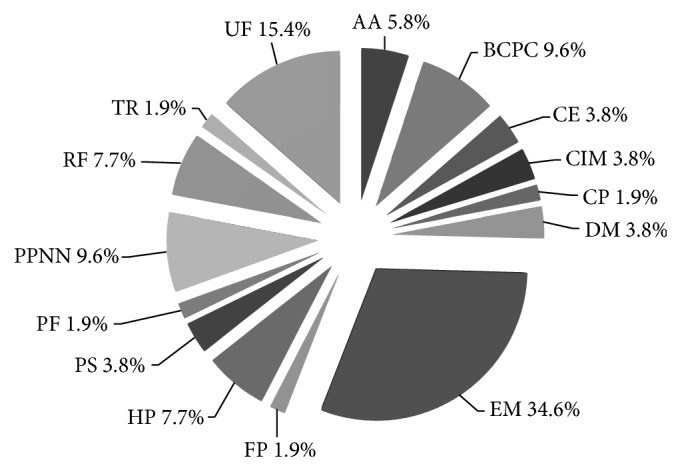
Functional classification of the differentially abundant proteins presented in Tables S1 and S2 shown as percentage of the total number of unique proteins. Functional categories are AA, amino acid metabolism; BCPC, biosynthesis of cofactors, prosthetic groups, and carriers; CIM, central intermediate metabolism; CE, cell envelope; CP, cellular processes; DM, DNA metabolism; EM, energy metabolism; FP, fatty acid and phospholipid metabolism; HP, hypothetical proteins; PF, protein fate; PPNN, purines, pyrimidines, nucleosides, and nucleotides; PS, protein synthesis; RF, regulatory functions; TR, transport; and UF, unknown function.

The identified 49 unique proteins found to change in abundance when NCFM was grown on cellobiose were distributed among 15 functional categories based on the Comprehensive Microbial Research (CMR) database (http://cmr.jcvi.org/) (Figure 2) as follows: 3 proteins associated with amino acid metabolism; 5 with biosynthesis of cofactors, prosthetic groups, and carriers; 2 with central intermediate metabolism; 2 with cell envelope; 1 with cellular processes; 2 with DNA metabolism; 18 with energy metabolism; 1 with fatty acid and phospholipid metabolism; 4 with hypothetical proteins; 2 with protein synthesis; 1 with protein fate; 5 with purines, pyrimidines, nucleosides, and nucleotides; 4 with regulatory functions; 1 with transport; and 8 of unknown function.

### 3.2. Differentially Expressed Proteins of NCFM Grown on Cellobiose

A major proportion of the proteins that changed in abundance were associated with energy metabolism (Figure 2), including enzymes involved in the glycolysis: triosephosphate isomerase (1.5–2.6-fold, spots 59, 60, 106, and 107, some being higher and some lower in their abundance), glyceraldehyde 3-phosphate dehydrogenase (1.5-2.5-fold, Table 1, spots 36–38, 70, and 123, some being higher and some lower in their abundance), and phosphoglycerate kinase (1.6-1.7-fold lower abundance, Table 1, spots 28–31). The two identified glycoside hydrolases, phospho-*β*-glucosidase and phospho-*β*-galactosidase II, were both upregulated. Phospho-*β*-glucosidase (Table 1, spot 17) increased 2.4-fold, whilst two forms of phospho-*β*-galactosidase II with different p*I* (Table 1, spots 16 and 18) increased 7.0- and 7.4-fold in NCFM grown on cellobiose as compared to glucose. Notably, trehalose-6-phosphate hydrolase (see Table S1 in Supplementary Material available online at http://dx.doi.org/10.1155/2015/347216, spot 93), catalyzing conversion of trehalose-6-phosphate to glucose and glucose 6-phosphate, was upregulated by 2.1-fold. Chromosome partitioning protein (Table S1, spot 48) and a putative oxalyl-CoA carboxylase (Table S1, spot 11) involved in both energy metabolism and central intermediate metabolism showed 3.1- and 1.9-fold lower abundance, respectively.

Differentially abundant proteins involved in protein synthesis including elongation factor Tu (EF-Tu) (Table S1, spots 25 and 27) decreased 4.2–6.5-fold, and a putative phosphate starvation inducible protein (Table S1, spot 72) increased 2.8-fold. Two forms of a putative serine protease (Table S1, spots 131 and 132) involved in protein fate showed a 1.5-1.6-fold decrease. Two isoforms of asparagine synthetase (Table S1, spots 33 and 136, and Table S2, spots 120 and 121) involved in amino acid metabolism increased 1.6–1.9-fold, while serine hydroxymethyltransferase (Table S1, spot 26), also associated with functional categories purines, pyrimidines, nucleosides, and nucleotides and biosynthesis of cofactors, prosthetic groups, and carriers, decreased 1.6-fold.

All identified proteins associated with purines, pyrimidines, nucleosides, and nucleotides were downregulated when grown on cellobiose compared to glucose. They include ribonucleoside triphosphate reductase (Table S1, spots 2–5), inosine-5′-monophosphate dehydrogenase (Table S1, spot 35), uracil* p*-ribotransferase (Table S2, spot 66), and 2′,3′-cyclic-nucleotide 2′-phosphodiesterase (Table 1, spot 92), which decreased 1.5–3.6-fold. Differentially expressed proteins involved in DNA metabolism were identified as DNA gyrase subunit B (Table S1, spot 6) and single-stranded DNA-binding protein (Table S1, spot 75) which increased 1.5- and 13.9-fold, respectively, when NCFM was grown on cellobiose.

The second largest group of proteins with altered abundancy was of unknown function (UF), which included three upregulated hypothetical proteins: LBA0466 (3.4- and 4.5-fold; Table S1, spots 7 and 8), LBA0890 (1.5–2.0-fold; Table S1, spots 54–56, 58, 109, and 124), and LBA1769 (2.0-fold; Table S1, spots 77 and 82). Other proteins in the so-called category of unknown function, that is, myosin-cross-reactive antigen (Table S1, spots 9 and 10), oxidoreductase (Table S1, spots 50 and 95), tRNA uridine 5-carboxymethylaminomethyl modification enzyme GidA (Table S1, spots 111, 112, and 114), and galactose mutarotase related enzyme (Table S1, spot 49), increased 1.6–6.3-fold. Proteins associated with prosthetic groups and carriers, that is, hypothetical protein LBA1769 (Table S1, spots 77 and 82), nicotinate phosphoribosyltransferase (Table S1, spot 23), and NAD synthetase (Table S1, spot 42), were upregulated by 1.7–2.0-fold, while aminotransferase (Table S1, spot 32) was 1.9-fold downregulated, as compared to growth in glucose. Among proteins involved in regulatory functions a two-component system regulator (Table 1, spot 102) was decreased 7.8-fold, while dihydroxyacetone kinase (Table S1, spot 43), transcriptional regulator (Table S1, spot 116), and catabolite control protein A (Table 1, spot 47) increased 2.4–4.2-fold. A putative surface layer protein (Table 1, spot 122) and UDP-*N-*acetylmuramoyl-L-alanyl-d-glutamate synthetase (Table S1, spot 15), both involved with cell envelope, were 2.2- and 1.5-fold higher, respectively, compared to cultures grown with glucose. Only one protein of the functional class involved in transport was observed to change, that is, an ABC transporter ATP-binding protein (Table S1, spots 113 and 117) which decreased 1.6-fold. Moreover, two forms of metallo-*β*-lactamase superfamily protein (Table S1, spots 115 and 125), presumably involved with cellular proteases, increased and decreased 2.2- and 2.0-fold, respectively. A single protein was identified from the fatty acid and phospholipid metabolism category: cyclopropane-fatty-acyl-phospholipid synthase (Table S1, spot 34), which showed 1.5-fold decreased abundance.

## 4. Discussion

Combining cellobiose with a probiotic strain of* Lactobacillus rhamnosus* was previously reported to give a synergistic increase of this and other lactic acid bacteria in the cecum of rats [33], but no proteins important for cellobiose utilization by lactic acid bacteria were identified in that study. The present work provides the first insights into the molecular mechanisms of the utilization of cellobiose by the probiotic* Lactobacillus acidophilus* NCFM. Differential proteome analysis by 2D-DIGE coupled with mass spectrometric protein identification highlighted enzymes and proteins important for uptake and catabolism of cellobiose in NCFM.

### 4.1. Proteins Involved in Carbohydrate Utilization and Regulatory Functions

Growth on cellobiose alters abundance of a large number of proteins of NCFM involved in glycolysis, some of which exist in multiple forms. This include proteins such as phosphoglucomutase found in two forms with different p*I* (Figure 1(c), spots 12 and 13); glyceraldehyde 3-phosphate dehydrogenase (GADPH) identified in multiple forms with different p*I* as well as molecular size (Figures 1(c) and 1(d), spots 36–38, 70, and 123); triosephosphate isomerase (TPI) present in four forms with different p*I* and molecular size (Figure 1(c), spots 59, 60, 106, and 107), and phosphoglycerate kinase found in four forms having different p*I* (Figure 1(c), spots 28–31). Multiple forms of phosphoglucomutase, GADPH, TPI, and phosphoglycerate kinase with different p*I* have previously been identified from NCFM [8]. Notably, multiple forms of GADPH and TPI were not affected in in the same way during growth on cellobiose.

**Figure 3:**
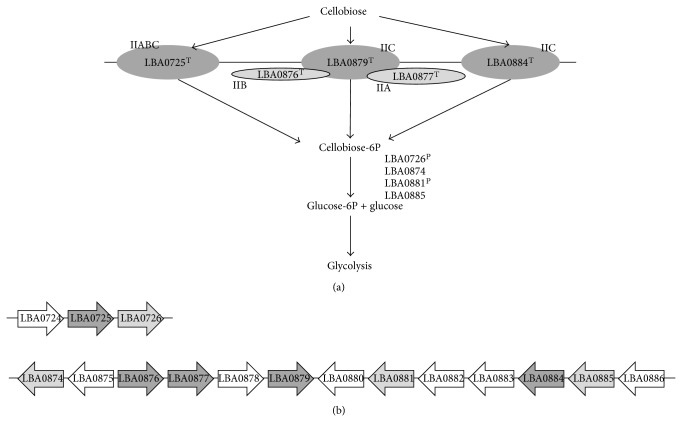
(a) Schematic presentation of cellobiose entry and hydrolysis by* L. acidophilus* NCFM. Superscript letters ^T^ and ^P^ indicate upregulation by transcriptomics [19] or increase in abundance by proteomics, respectively (LBA0725: PTS II, LBA0726: phospho-*β*-galactosidase II, LBA0874: phospho-*β*-galactosidase I, LBA0876: PTS IIC, LBA0877: PTS IIA, LBA0879: PTS IIC, LBA0881: phospho-*β*-glucosidase, LBA0884: PTS IIC LBA0726: phospho-*β*-galactosidase II, and LBA0885: *β*-glucosidase). (b) Schematic presentation of gene clusters encoding glycoside hydrolases (LBA0726: phospho-*β*-galactosidase II, LBA0874: phospho-*β*-galactosidase I, LBA0881: phospho-*β*-glucosidase, and LBA0885: *β*-glucosidase), shown as light grey arrows and PTSs (LBA0725: PTS II, LBA0876: PTS IIC, LBA0877: PTS IIA, LBA0879: PTS IIC, and LBA0884: PTS IIC), shown as dark grey arrows, predicted to be cellobiose specific. Transcription antiterminator (LBA0724), hypothetical proteins (LBA0878, LBA0880, and LBA0883), and transcriptional regulators (LBA0875, LBA0882, and LBA0886) are shown as white arrows.

Growth of NCFM on cellobiose elicited an increase in abundance of catabolite control protein A (CcpA; Table 1, spot 47). CcpA is a key enzyme in carbon catabolite repression, a mechanism that regulates enzymes involved in carbohydrate metabolism. Thus in the presence of preferred sugars (e.g., glucose) enzymes involved in utilization of less favorable sugars are repressed [34]. Carbohydrate phosphotransferase systems (PTSs) involved in uptake and phosphorylation of carbohydrates participate in the carbon catabolite repression [34]. When NCFM was grown on cellobiose, notably 2.4-fold upregulation was seen for phospho-*β*-glucosidase (LBA0881; Table 1, spot 17) located in a gene cluster with multiple PTSs predicted to be cellobiose specific (Figure 3). Interestingly a 7.0–7.4-fold increased abundance was observed for phospho-*β*-galactosidase II (LBA0726; Table 1, spots 16 and 18) encoded in a gene cluster together with another PTS (LBA0725; Figure 3) indicating a role in cellobiose utilization (Figure 3). This PTS LBA0725 has been reported to have 62% amino acid sequence identity to a PTS (ORF 1669) of* L. gasseri* 33323 of which expression was strongly induced by cellobiose [35]. In another low GC ratio, Gram-positive bacteria,* Clostridium acetobutylicum*, transcriptional analysis demonstrated that cellobiose induced expression of two PTSs and three glycoside hydrolases [25]. Transcriptional analysis of NCFM grown on cellobiose has reported upregulation of several genes within the loci LBA0724–LBA0726 and LBA0877–LBA0884 [26]. Taken together, the results of transcriptional and proteomics analyses indicate that NCFM contains more than one operon encoding protein involved in uptake and metabolism of cellobiose.

NCFM encodes 9 two-component system regulators (2CSRs) [7] involved in mediating an intracellular response to extracellular stimuli and typically consisting of a membrane bound histidine protein kinase (HPK) and a cytoplasmic response regulator (RR) [36]. When NCFM was grown on cellobiose a 2CSR (Table 1, spot 102) was found to be downregulated by 7.8-fold. It has been reported that a 2CRS is involved in acid tolerance and proteolytic activity of NCFM [37]. The decrease in abundance of the 2CSR, however, may reflect the different growth rates of NCFM on cellobiose and glucose rather than a decrease in acid tolerance or proteolytic activity. This could also explain why all the proteins with changed abundance in the functional categories of protein synthesis, central intermediate metabolism, and purines, pyrimidines, nucleosides, and nucleotides, apart from a putative phosphate starvation inducible factor, were less abundant in the cellobiose-grown NCFM. Moreover, two different subunits of the F_0_F_1_ ATP synthase (Table 1, spots 41 and 99), responsible for maintaining the proton motive force at low pH [36], decreased by 1.6- and 1.9-fold, respectively, when NCFM was grown on cellobiose. These various changes in the protein profile are in agreement with the observed slower growth of NCFM on cellobiose compared to glucose.

Several proteins previously described as moonlighting proteins were identified. These include EF-Tu, GAPDH, and TPI with conventional roles in protein synthesis and glycolysis, respectively, but which also have been identified at the cell wall where they are involved in adhesion activities of lactic acid bacteria [38–41]. Although the present study includes only differentially expressed cytosolic proteins, a putative surface layer protein was also identified and found to be upregulated (Table 1, spot 122). Frece et al. [42] reported that removing the S-layer of* L. acidophilus* M92, by enzymatic and chemical treatment, resulted in reduced adhesion to murine epithelial cells and reduced viability. Moreover, S-layer proteins of* Lactobacillus crispatus* are able to inhibit the adhesion of pathogens to HeLa cells [43]. In NCFM, S-layer protein A was shown to bind to a dendritic cell receptor and to regulate immune functions of the dendritic cells [44]. The putative S-layer protein upregulated in the present study has recently been shown to be an S-layer associated [45]. Although a deletion mutant of this S-layer associated protein did not affect adhesion to intestinal cells, a reduction of proinflammatory response in murine dendritic cells was observed [45] thus suggesting an effect on the host immune system. The present findings propose that cellobiose is able to affect the probiotic activity of NCFM with regard to interactions with the host.

## 5. Conclusions

Exploring the molecular mechanisms of prebiotic-probiotic interactions is important for understanding how prebiotics benefit a probiotic strain. In the current study, the late exponential growth phase differential proteome of* L. acidophilus* NCFM (pH 4–7 and pH 6–11) was explored using the disaccharide cellobiose as carbon source. Cellobiose consists of two *β*(1→4) linked glucosyl residues which after hydrolysis enter the glycolysis. Two hydrolases, a phospho-*β*-glucosidase (LBA0881; Table 1, spot 17) and a phospho-*β*-galactosidase II (LBA0726; Table 1, spots 16 and 18), increased 2.4–7.4-fold in abundance, suggesting an involvement in the cellobiose metabolism (Figure 3). Results obtained in the present study contribute to the understanding of prebiotic utilization by probiotic bacteria.

## Supplementary Material

Table S1. Differentially abundant proteins identified by peptide mass fingerprint of *Lactobacillus acidophilus* NCFM grown on cellobiose compared to growth on glucose. Differential abundance was based on ProgenesisSameSpots analyses of 2D images (>1.5-fold spot volume ratio change; ANOVA p ≤ 0.05 and false discovery rate q <0.05). A Mascot score of ≥ 80 (p ≤ 0.05) was used to confirm proteins identified and should have a minimum of six matched peptides. Proteins are listed according to their fold change.
Table S2. Differentially abundant proteins, confirmed by MS/MS, of *Lactobacillus acidophilus* NCFM grown on cellobiose compared to glucose. Differential abundance was based on ProgenesisSameSpots analyses of 2D images (>1.5-fold spot volume ratio change; ANOVA p ≤ 0.05 and false discovery rate q <0.05). MS/MS was confirmed by a Mascot score of ≥ 40 (p ≤ 0.05) for each peptide. Proteins are listed according to their fold change.

## Figures and Tables

**Table 1:** Differentially abundant proteins of *Lactobacillus  acidophilus* NCFM grown on cellobiose compared to growth on glucose shown for selected proteins having a role in carbohydrate metabolism. Differential abundance was based on Progenesis SameSpots analyses of 2D images (≥ 1.5-fold spot volume ratio change; ANOVA *P* ≤ 0.05 and false discovery rate *q* ≤ 0.05). A Mascot score of ≥ 80 (*P* ≤ 0.05) was used to confirm proteins identified by peptide mass fingerprint and should have a minimum of six matched peptides. Proteins are listed according to their fold change. All identified proteins including information regarding score, ANOVA, sequence coverage, peptide search and identification, *MW*, and p*I* are available in supplementary material.

Spot number	Fold change	Accession number	Protein name	Localization^a^	Functional role^b^
^*^102	−7.8	gi∣58337790	Two-component system regulator	C	RF
16	+7.4	gi∣58337043	Phospho-*beta*-galactosidase II	C	EM
18	+7.0	gi∣58337043	Phospho-*beta*-galactosidase II	C	EM
92	−3.6	gi∣58337251	2′,3′-Cyclic-nucleotide 2′-phosphodiesterase	E	PPNN
13	+3.5	gi∣58337008	Phosphoglucomutase	C	EM
60	+2.6	gi∣58337021	Triosephosphate isomerase	C	EM
37	−2.5	gi∣58337019	Glyceraldehyde-3P dehydrogenase	C	EM
17	+2.4	gi∣58337186	Phospho-*beta*-glucosidase	C	EM
47	+2.4	gi∣58336768	Catabolite control protein A	C	RF
122	+2.2	gi∣58337323	Putative surface layer protein	CW	CE
123	+2.0	gi∣58337019	Glyceraldehyde-3P dehydrogenase	C	EM
99	−1.9	gi∣58337088	F0F1 ATP synthase subunit alpha	C	EM
59	−1.8	gi∣58337021	Triosephosphate isomerase	C	EM
29	−1.7	gi∣58337020	Phosphoglycerate kinase	C	EM
106	−1.7	gi∣58337021	Triosephosphate isomerase	C	EM
28	−1.6	gi∣58337020	Phosphoglycerate kinase	C	EM
30	−1.6	gi∣58337020	Phosphoglycerate kinase	C	EM
31	−1.6	gi∣58337020	Phosphoglycerate kinase	C	EM
38	−1.6	gi∣58337019	Glyceraldehyde-3P dehydrogenase	C	EM
41	−1.6	gi∣58337089	F0F1 ATP synthase subunit gamma	C	EM
36	+1.5	gi∣58337019	Glyceraldehyde-3P dehydrogenase	C	EM
70	−1.5	gi∣58337019	Glyceraldehyde-3P dehydrogenase	C	EM
107	−1.5	gi∣58337021	Triosephosphate isomerase	C	EM

^a^Localization: C, cytoplasmic; CW, cell wall; E, extracellular.

^b^Functional role: CE, cell envelope; EM, energy metabolism; PPNN, purines, pyrimidines, nucleosides, and nucleotides; RF, regulatory functions.

^*^Protein identification by MS/MS was confirmed by a Mascot score of ≥ 40 (*P* ≤ 0.05) for each peptide.

## List of Abbreviations

2CSR: Two-component system regulator
CcpA: Catabolite control protein A
CHAPS: 3-((3-Cholamidopropyl)dimethylammonio)-1-propansulfonate
GAPDH: Glyceraldehyde 3-phosphate dehydrogenase
GH: Glycoside hydrolase
HED: Bis(2-hydroxyethyl) disulphide
HPK: Histidine protein kinase
HPr: Histidine containing phosphocarrier protein
IEF: Isoelectric focusing
IPG: Immobilized pH gradient
LABSEM: Lactic acid bacteria semisynthetic medium
NCFM: * Lactobacillus acidophilus* NCFM
PTS: Phosphotransferase system
RR: Response regulator
SCFA: Short-chain fatty acid
TPI: Triosephosphate isomerase.
