# Supplementary material for: The Differential Proteome of the Probiotic Lactobacillus acidophilus NCFM Grown on the Potential Prebiotic Cellobiose Shows Upregulation of Two β-Glycoside Hydrolases
Source: Biomed Res Int. 2015 Apr 19;2015:347216. doi: 10.1155/2015/347216 (PMC4417578; doi:10.1155/2015/347216)
Supplement: Supplementary file 1 — Table S1. Differentially abundant proteins identified by peptide mass fingerprint of Lactobacillus acidophilus NCFM grown on cellobiose compared to growth on glucose. Differential abundance was based on ProgenesisSameSpots analyses of 2D images (>1.5-fold spot volume ratio change; ANOVA p ≤ 0.05 and false discovery rate q <0.05). A Mascot score of ≥ 80 (p ≤ 0.05) was used to confirm proteins identified and should have a minimum of six matched peptides. Proteins are listed according to their fold change. Table S2. Differentially abundant proteins, confirmed by MS/MS, of Lactobacillus acidophilus NCFM grown on cellobiose compared to glucose. Differential abundance was based on ProgenesisSameSpots analyses of 2D images (>1.5-fold spot volume ratio change; ANOVA p ≤ 0.05 and false discovery rate q <0.05). MS/MS was confirmed by a Mascot score of ≥ 40 (p ≤ 0.05) for each peptide. Proteins are listed according to their fold change. [file 347216.f1.pdf]

Table S1.

Spot no.	Fold change	Accession no.	Protein name	Score	ANOVA	Sequence coverage	Peptides matched/seached	MW/pI	Localization[a]	Functional role[b]
75	+ 13.9	gi|58336362	single-stranded DNA-binding protein	126	2.60E-06	43%	8/22	18932/4.96	U	DM
16	+ 7.4	gi|58337043	phospho-beta-galactosidase II	120	1.00E-05	19%	12/32	56703/5.04	C	EM
18	+ 7.0	gi|58337043	phospho-beta-galactosidase II	135	3.30E-07	23%	14/41	56703/5.04	C	EM
25	- 6.5	gi|58337152	elongation factor Tu	166	2.60E-10	56%	19/48	43609/4.97	C	PS
9	+ 6.3	gi|58336882	myosin-crossreactive antigen	96	2.40E-03	15%	8/13	67700/5.91	U	UF
20	+ 6.2	gi|58337725	galactose-1-phosphate uridylyltransferase	105	1.60E-04	22%	10/20	55735/5.48	C	EM
10	+ 5.5	gi|58336882	myosin-crossreactive antigen	129	1.30E-06	25%	13/30	67700/5.91	U	UF
8	+ 4.5	gi|58336799	hypothetical protein LBA0466	132	6.60E-07	21%	10/15	63103/5.39	C	EM; HP; UF
95	+ 4.4	gi|58337322	oxidoreductase	117	9.80E-06	41%	10/37	31816/5.19	C	UF
27	- 4.2	gi|58337152	elongation factor Tu	157	2.10E-09	60%	18/85	43609/4.97	C	PS
43	+ 4.2	gi|58337704	dihydroxyacetone kinase	123	5.20E-06	42%	12/51	36307/5.16	C	RF
2	- 4.0	gi|58336392	ribonucleoside triphosphate reductase	148	7.80E-09	27%	18/45	83982/5.62	C	PPNN
50	+ 3.9	gi|58337322	oxidoreductase	115	3.30E-05	37%	9/30	31816/5.19	C	UF
92	- 3.6	gi|58337251	2',3'-cyclic-nucleotide 2'-phosphodiesterase	82	3.20E-02	21%	12/57	59124/5.68	E	PPNN
13	+ 3.5	gi|58337008	phosphoglucomutase	97	2.20E-03	29%	14/50	64122/5.18	C	EM
7	+ 3.4	gi|58336799	hypothetical protein LBA0466	100	1.10E-03	18%	7/9	63103/5.39	C	EM; HP; UF
21	+ 3.2	gi|58337891	NAD-dependent aldehyde dehydrogenase	90	1.10E-02	22%	9/26	50588/5.14	C	EM
48	- 3.1	gi|58338075	chromosome partitioning protein	103	5.20E-04	33%	7/14	28414/5.87	C	CIM; EM
52	+ 3.1	gi|58336369	fructokinase	109	1.30E-04	45%	11/52	32016/5.08	C	EM
22	- 3.0	gi|58337891	NAD-dependent aldehyde dehydrogenase	124	4.10E-06	40%	14/43	50588/5.14	C	EM
72	+ 2.8	gi|58336994	putative phosphate starvation inducible protein stressrelated	127	2.10E-06	55%	9/20	21503/5.46	C	PS
44	- 2.7	gi|58336405	D-lactate dehydrogenase	156	2.60E-09	58%	16/52	39177/4.96	C	EM
60	+ 2.6	gi|58337021	triosephosphate isomerase	98	1.50E-03	41%	8/32	27770/4.72	C	EM
116	+ 2.6	gi|58337187	transcriptional regulator	134	2.00E-07	38%	9/13	28083/7.82	C	RF

12	+ 2.5	gi|58337008	phosphoglucomutase	105	3.30E-04	17%	9/14	64122/5.18	C	EM
37	- 2.5	gi|58337019	glyceraldehyde-3-p dehydrogenase	177	2.10E-11	53%	17/48	36643/5.92	C	EM
17	+ 2.4	gi|58337186	phospho-beta-glucosidase	118	1.60E-05	23%	10/17	56111/5.28	C	EM
47	+ 2.4	gi|58336768	catabolite control protein A	155	3.30E-09	50%	14/35	37154/5.43	C	RF
112	+ 2.3	gi|58338214	tRNA uridine 5-carboxymethylaminomethyl modification enzyme GidA	98	7.50E-04	25%	14/41	70719/6.99	C	UF
40	+ 2.2	gi|58337213	L-lactate dehydrogenase	96	2.70E-03	43%	8/42	33442/4.95	C	EM
115	+ 2.2	gi|58337136	metallo-b-lactamase superfamily protein	103	2.50E-04	21%	9/24	61888/6.43	C	CP; UF
122	+ 2.2	gi|58337323	putative surface layer protein	128	7.80E-07	20%	11/22	42761/9.74	CW	CE
66	- 2.1	gi|58337083	uracil p-ribosyltransferase	135	3.30E-07	51%	9/15	22918/5.36	C	PPNN
93	+ 2.1	gi|58337309	trehalose 6-P hydrolase	96	2.50E-03	27%	16/50	64668/5.7	C	EM
111	+ 2.1	gi|58338214	tRNA uridine 5-carboxymethylaminomethyl modification enzyme GidA	127	9.80E-07	28%	16/34	70719/6.99	C	UF
58	+ 2.0	gi|58337488	hypothetical protein LBA0890	116	2.60E-05	36%	10/33	31352/5.15	C	HP; UF
77	+ 2.0	gi|58338023	hypothetical protein LBA1769	118	1.60E-05	68%	8/30	13558/5.65	C	BCPC; HP
82	+ 2.0	gi|58338023	hypothetical protein LBA1769	136	2.60E-07	66%	8/17	13558/5.65	C	BCPC; HP
123	+ 2.0	gi|58337019	glyceraldehyde-3-p dehydrogenase	80	4.50E-02	17%	6/26	36643/5.92	C	EM
125	- 2.0	gi|58337136	metallo-b-lactamase superfamily protein	141	3.90E-08	21%	10/20	61888/6.43	C	CP; UF
11	- 1.9	gi|58336733	putative oxalyl-CoA decarboxylase	184	4.10E-12	28%	19/27	60969/6.03	CM	CIM; EM
32	- 1.9	gi|58337100	aminotransferase	124	4.10E-06	26%	8/12	42549/5.51	C	BCPC
33	+ 1.9	gi|58338140	asparagine synthetase AsnA	116	2.60E-05	39%	11/26	38890/5.84	C	AA
99	- 1.9	gi|58337088	F0F1 ATP synthase subunit alpha	188	7.80E-13	48%	23/72	54956/4.94	C	EM
114	+ 1.9	gi|58338214	tRNA uridine 5-carboxymethylaminomethyl modification enzyme GidA	82	3.40E-02	16%	10/20	70719/6.99	C	UF
124	+ 1.9	gi|58337195	hypothetical protein LBA0890	86	1.20E-02	27%	8/21	30855/6.32	C	HP; UF
59	- 1.8	gi|58337021	triosephosphate isomerase	177	2.10E-11	53%	14/44	27770/4.72	C	EM
23	+ 1.7	gi|58336848	nicotinate phosphoribosyltransferase	159	1.30E-09	30%	15/30	56238/5.35	C	BCPC
29	- 1.7	gi|58337020	phosphoglycerate kinase	186	2.60E-12	60%	21/74	42849/5.19	C	EM
35	- 1.7	gi|58336540	inosine-5'-monophosphate dehydrogenase	157	2.10E-09	39%	13/30	39816/5.75	C	PPNN
42	+ 1.7	gi|58336849	NAD synthetase	124	2.00E-06	56%	12/62	31118/5.09	C	BCPC

106	- 1.7	gi|58337021	triosephosphate isomerase	141	3.90E-08	53%	14/48	27770/4.72	C	EM
109	+ 1.7	gi|58337195	hypothetical protein LBA0890	215	1.60E-15	61%	19/32	30855/6.32	C	UF
4	- 1.6	gi|58336392	ribonucleoside triphosphate reductase	164	4.10E-10	32%	21/57	83982/5.62	C	PPNN
5	- 1.6	gi|58336392	ribonucleoside triphosphate reductase	191	8.30E-13	33%	22/50	83982/5.62	C	PPNN
26	- 1.6	gi|58336600	serine hydroxymethyltransferase	108	1.60E-04	20%	11/31	45276/5.56	C	AA; BCPC; PPNN
28	- 1.6	gi|58337020	phosphoglycerate kinase	159	1.30E-09	52%	19/82	42849/5.19	C	EM
30	- 1.6	gi|58337020	phosphoglycerate kinase	201	8.30E-14	71%	24/87	42849/5.19	C	EM
31	- 1.6	gi|58337020	phosphoglycerate kinase	164	4.10E-10	39%	13/25	42849/5.19	C	EM
38	- 1.6	gi|58337019	glyceraldehyde-3-p dehydrogenase	153	5.20E-09	57%	15/46	36643/5.92	C	EM
41	- 1.6	gi|58337089	F0F1 ATP synthase subunit gamma	97	2.10E-03	26%	9/24	35512/5.93	C	EM
46	- 1.6	gi|58336610	L-LDH	153	5.20E-09	58%	15/55	35074/5.06	C	EM
49	+ 1.6	gi|58337283	galactose mutarotase related enzyme	130	1.00E-06	56%	13/52	34719/5.22	C	UF
54	+ 1.6	gi|58337195	hypothetical protein LBA0890	87	2.30E-02	26%	7/15	30855/6.32	C	HP; UF
56	+ 1.6	gi|58337195	hypothetical protein LBA0890	99	1.40E-03	31%	8/18	30855/6.32	C	HP; UF
113	- 1.6	gi|58338086	ABC transporter ATP-binding protein	91	4.30E-03	46%	10/64	24611/9.24	C	TR
117	- 1.6	gi|58338086	ABC transporter ATP-binding protein	139	6.20E-08	60%	13/40	24611/9.24	C	TR
131	- 1.6	gi|58337841	putative serine protease	121	3.90E-06	30%	17/43	78332/9.54	CW/C	PF
136	+ 1.6	gi|58336505	asn synthetase	94	1.80E-03	19%	11/24	75771/6.47	C	AA
3	- 1.5	gi|58336392	ribonucleoside triphosphate reductase	150	4.90E-09	26%	17/38	83982/5.62	C	PPNN
6	+ 1.5	gi|58336359	DNA gyrase subunit B	156	2.60E-09	24%	12/15	72984/5.82	C	DM
15	+ 1.5	gi|58337117	UDP-N-acetylmuramoyl-L-alanyl-D-glutamate synthetase	109	1.30E-04	26%	11/30	51180/5.24	C	CE
34	- 1.5	gi|58337550	cyclopropane-fatty-acyl-phospholipid synthase	100	1.00E-03	24%	7/13	45345/5.44	C	FP
36	+ 1.5	gi|58337019	glyceraldehyde-3-p dehydrogenase	209	1.30E-14	65%	19/53	36643/5.92	C	EM
55	+ 1.5	gi|58337195	hypothetical protein LBA0890	151	8.30E-09	36%	10/15	30855/6.32	C	HP; UF
70	- 1.5	gi|58337019	glyceraldehyde-3-p dehydrogenase	84	4.00E-02	22%	7/22	36643/5.92	C	EM
107	- 1.5	gi|58337021	triosephosphate isomerase	98	7.50E-04	47%	9/30	27770/4.72	C	EM
132	- 1.5	gi|58337841	putative serine protease	85	1.70E-02	27%	17/70	78332/9.54	CW/C	PF

[a] Localization: C, cytoplasmic; CM, cytoplasmic membrane, CW, cell wall; E, extracellular; U, unknown

[b] Functional role: AA - Amino acid metabolism; BCPC - Biosynthesis of cofactors; prosthetic groups, and carriers; CE - Cell envelope; CIM - Central intermediary metabolism; CP - Cellular processes; DM - DNA metabolism; EM - Energy metabolism; FP - Fatty acid and phospholipid metabolism; HP - Hypothetical proteins; PS - Protein synthesis; PF - Protein fate; PPNN - Purines, pyrimidines, nucleosides and nucleotides; RF - Regulatory functions; TR – Transport; UF - Unknown functions

Table S2.

Spot no.	Fold change	Accession no.	Protein name	Sequence of peptides identified by MS/MS	Score	ANOVA	MW/pI	Localization[a]	Functional role[b]
102	- 7.8	gi|58337790	two-component system regulator	ILIIEDEKNLAR	53	2.20E-02	27227/5.61	C	RF
				FKDLTIETANR	60	6.10E-03			
120	+ 1.8	gi|58336505	asn synthetase	DANSILQPTYR	50	3.50E-02	75771/6.47	C	AA
					103	2.50E-04			
121	+ 1.8	gi|58336505	asn synthetase	DANSILQPTYR	52	3.10E-02	75771/6.47	C	AA

See Table S1 for footnotes.
